# Role of High-Fat Diet in Stress Response of *Drosophila*

**DOI:** 10.1371/journal.pone.0042587

**Published:** 2012-08-01

**Authors:** Erilynn T. Heinrichsen, Gabriel G. Haddad

**Affiliations:** 1 Department of Pediatrics (Division of Respiratory Medicine) and Biomedical Sciences Graduate Program, University of California San Diego, La Jolla, California, United States of America; 2 Department of Neurosciences, University of California San Diego, La Jolla, California, United States of America; 3 Rady Children's Hospital, San Diego, California, United States of America; Queen Mary University of London, United Kingdom

## Abstract

Obesity is associated with many diseases, one of the most common being obstructive sleep apnea (OSA), which in turn leads to blood gas disturbances, including intermittent hypoxia (IH). Obesity, OSA and IH are associated with metabolic changes, and while much mammalian work has been done, mechanisms underlying the response to IH, the role of obesity and the interaction of obesity and hypoxia remain unknown. As a model organism, *Drosophila* offers tremendous power to study a specific phenotype and, at a subsequent stage, to uncover and study fundamental mechanisms, given the conservation of molecular pathways. Herein, we characterize the phenotype of *Drosophila* on a high-fat diet in normoxia, IH and constant hypoxia (CH) using triglyceride and glucose levels, response to stress and lifespan. We found that female flies on a high-fat diet show increased triglyceride levels (p<0.001) and a shortened lifespan in normoxia, IH and CH. Furthermore, flies on a high-fat diet in normoxia and CH show diminished tolerance to stress, with decreased survival after exposure to extreme cold or anoxia (p<0.001). Of interest, IH seems to rescue this decreased cold tolerance, as flies on a high-fat diet almost completely recovered from cold stress following IH. We conclude that the cross talk between hypoxia and a high-fat diet can be either deleterious or compensatory, depending on the nature of the hypoxic treatment.

## Introduction

Over 60% of the population in the United States is estimated to be obese or overweight, a number that has dramatically increased in recent decades and continues to climb [1]. A multitude of social and economic factors have contributed to the rise in obesity, not the least of which is an abundance of processed foods high in saturated fat and simple carbohydrates. With obesity comes many disease complications, including sleep apnea, hypoxia, atherosclerosis, cardiovascular diseases and stroke [2].

In obstructive sleep apnea (OSA), one of the most common associated diseases, the upper airway collapses repeatedly during sleep, causing chronic intermittent hypoxia [3]. OSA patients therefore are often challenged with both obesity and intermittent hypoxia conditions. In order to investigate the potential interaction of obesity and hypoxia, we needed a model that would allow us to study metabolic changes and be able to survive hypoxic challenges. Furthermore, such a model would need to lend itself to molecular analysis to appreciate the basis for such phenotypic changes.

The fruit fly, *Drosophila melanogaster*, has served as a useful genetic model system in many situations, including development and human disease states. We undertook this investigation to determine whether we can develop a model in the fly that would be helpful for understanding the effects of hypoxia and obesity and their potential interactions. We believe that the fly could be an important model for various reasons, including the facts that a) many of the metabolic and signaling pathways involved in fat metabolism and insulin signaling in flies are conserved in humans [4] and b) *Drosophila* have many organ systems analogous to humans that control uptake, storage and metabolism; one of these is the fat body, which functions like human liver and white adipose tissue, metabolizing nutrients and storing reserves of glycogen and lipids [4]. The adult fat body is subject to diet-induced lipid overload, making *Drosophila* even more appealing as a genetic model in which to study obesity [5]–[7]. Furthermore, many human disease genes (>70%) have been found to exist in flies [8].

Since the early 1990s, our laboratory has used *Drosophila melanogaster* to investigate the effects of hypoxia on metabolism, gene regulation, and the role of gene regulation in resistance or susceptibility to injury. More recently, we have studied the effects of both intermittent (IH) and constant hypoxia (CH) [9]–[11], since each of these conditions occurs in many different diseases and causes significant stress on the organism. Additionally, we have shown that the two separate paradigms of IH and CH result in a differential change in gene expression [12], making it all the more important to individually evaluate and compare the two stresses.

With the ability to put flies on a diet high in saturated fat while simultaneously exposing them to either normoxic or hypoxic conditions, we were able to investigate three different aims. These involved assessing the effects of a high-fat diet, the effects of intermittent hypoxia, and their potential interaction on the phenotype of *Drosophila*. We hypothesized that intermittent hypoxia alters lipid metabolism, leading to changes in stress tolerance in *Drosophila*, and the results show clear evidence of this interaction.

## Materials and Methods

### Fly Rearing and Collection

All stocks were maintained on standard cornmeal *Drosophila* medium in an incubator at 25°C and 30–50% humidity. Adult flies were collected at 0–3 days and transferred to a separate vial of the standard cornmeal medium in room air. After aging for 3 more days, male and female flies were separated and only the females were transferred to the experimental diet and oxygen condition. In all experiments, unless otherwise noted, *w^1118^* flies were used in order to facilitate future work with genetically mutated lines, since this is a common background of flies with P-element and UAS insertions. When *adipose^60^* mutants were studied, the wildtype strain used was *Oregon-R* in order to control for genetic background. The stocks of *Oregon-R*, *Canton-S* and *w^1118^* were obtained from Bloomington Stock Center, while *adipose^60^* was a gift from Dr. Tania Reis.

### Experimental Diets: Regular food and Coconut food

Jazz Mix *Drosophila* food from New Horizon Foods was prepared as directed and placed in plastic vials as the Regular food (RF). The Coconut food (CF) diet was based on a recipe developed by Dr. Sean Oldham at the Sanford/Burnham Institute, adding coconut oil to the regular food as a source for increased saturated fat in the diet [7]. The recipe has been specialized for the current model, with the coconut food diet consisting of 5, 10 or 20% weight per volume of food-grade coconut oil, with the last two higher than the maximum 7% saturated fat recommended by the American Heart Association. The 5% diet did not have an obvious enough effect, and no significant difference was found between the 10 and 20% supplemented food, so only results from the 20% diets are reported. For verification of results, we also tested a diet supplemented with palm oil in the same manner as described above for coconut oil.

### Experimental Oxygen Conditions: Normoxia, Intermittent and Constant Hypoxia

At age 3–6 days, female flies were sorted onto the diets and immediately placed in normoxia, intermittent hypoxia or constant hypoxia, with approximately 25 flies per vial. All three conditions were at room temperature (22–24°C) and in a similar environment.

In normoxic conditions, flies were kept in room air (21% O_2_). For intermittent hypoxia (IH) and constant hypoxia (CH), flies were placed in specially designed chambers where the oxygen levels are carefully controlled using a combination of oxygen and nitrogen with the Oxycycler hydraulic system (Model A44×0, BioSpherix, Redfield, NY) and ANA-Win2 Software (Version 2.4.17, Watlow Anafaze, CA). In the chamber for IH, flies were exposed to O_2_ levels alternating between 4 minutes at 1% and 4 minutes at 21% O_2_. The total time for one IH cycle was 20 minutes, with a ramp time of 1 minute for 1%–21% O_2_ and around 10 minutes for 21%–1% O_2_. In the CH chamber, flies were exposed to a constant oxygen level of 5% O_2_. Oxygen levels were chosen based on previous observations in our laboratory in order to allow flies to be mobile and still consume their diets.

Flies remained in their oxygen condition for one week if they were to be assayed or as long as needed to examine lifespan. To maintain consistent food conditions, flies were transferred to fresh food of their respective diet every 3–4 days.

### Metabolic Profile: Triglyceride, Glucose and Protein Measurement

After one week in a specific oxygen condition, flies were collected in groups of five female flies and placed in 1.5 ml microcentrifuge tubes. Their live weight was determined and the flies were frozen on dry ice. They were then homogenized using the Precelly's 24 homogenizer and prepared as described in Grönke 2003 [13] to measure absolute triglyceride levels using the Thermo Infinity Triglyceride kit and protein levels using Pierce BCA protein assay (protein levels not shown, as normalizing triglycerides to protein was comparable with normalizing to bodyweight). Differing levels of triglycerides reported in the current literature can easily be accounted for by multiple factors, including the sex and age of the flies, and the method of extraction. To determine whole-body glucose, groups of five female flies were homogenized in 1 ml deionized water. The homogenates were centrifuged and the supernatants transferred to a 96-well plate in triplicate. Glucose levels were quantified using the Glucose GO assay kit (Sigma–Aldrich, Saint Louis, MO) according to the manufacturer's instructions. In brief, glucose is oxidized to gluconic acid and hydrogen peroxide by glucose oxidase. The hydrogen peroxide then reacts with o-dianisidine in the presence of peroxide to form a colored product, and the oxidized o-dianisidine reacts with sulfuric acid to form a more stable colored product. The intensity of the color is proportional to the original glucose concentration. The whole-body free glucose was measured, although it is important to note that the major carbohydrate in *Drosophila* is trehalose. Trehalose is the product of two glycolytic intermediates, with the condensation of glucose-6-phosphate and the glucose moiety of uridine diphosphoglucose (UDP-glucose). Trehalose is then hydrolyzed into glucose by the enzyme trehalase. This study measures the glucose already available in the organism.

### CAFE Assay

Flies were placed on regular food (RF) and in normoxia or IH for 5 days. Following that time, groups of 5 flies were placed in a plastic vial with only a piece of filter paper containing 500 µl water. Through the top plug, a capillary tube was inserted containing 5 µl liquid food (5% yeast, 5% sucrose) as described in Ja et al [14]. The capillary tube was removed every 24 hours and replaced with a new capillary tube containing 5 µl of food. The flies were allowed to adjust to the new setup for the initial 24 hours, after which measurements were taken each time the capillary was removed, measuring the difference in level of food (in mm). Knowing the initial height (in mm) of the 5 µl, the calculated µl/mm could be multiplied by the change in food level to determine how much food (in µl) was consumed.

### Stress Tolerance: Cold, Anoxia and Starvation

All assays were performed in normoxia, immediately following the week of exposure to experimental diet and oxygen conditions. Flies exposed to the different diets were assayed simultaneously when possible (cold and starvation) or consecutively (anoxia).

#### Cold Stress

A −5°C bath was made using water, ice and salt. For each group, sets of 15–20 female flies were placed in empty plastic vials and into the water bath. Flies fell unconscious almost immediately and vials were checked to make sure all flies were at the bottom and thus submerged in the cold bath. They remained as such for 2 hours, with the temperature being checked regularly throughout. At the end of the 2 hours, vials were removed from the water bath, and flies transferred to regular food and left to recover at room temperature. After 24 hours, survival was recorded as the number of flies that had regained consciousness.

#### Anoxic Stress

A specially designed chamber was used to study flies under controlled O_2_ levels [9]. Sets of 30 flies from a particular group were placed in the chamber and exposed to anoxic conditions (O_2_ concentration = 0% with administration of 100% N_2_) for 2 hours. They were then returned to a vial with regular food in room air, and the number of flies that regained consciousness after 24 hours of recovery was counted as the survival.

#### Starvation

For each group, sets of 10–15 female flies were placed in a plastic vial with no food. A small circular filter paper was placed in the bottom of the vial with 75 ul of water to prevent dehydration and this was replenished with water every 16 hours or as needed. Survival was recorded every 4–8 hours as the number of flies alive in each vial.

### Lifespan

Flies were placed on experimental diets and in oxygen conditions as described for previous assays. The lifespan of the flies was observed by recording the number of flies alive each day in that oxygen condition. Flies were transferred to fresh food every 3–4 days. The experiment was concluded when there were no flies remaining in a group or when one group was below 50% survival and there was a clear difference between groups.

### Statistical Analysis

Graphpad Prism was used for statistical analysis. A t-test was used to determine significance between RF and CF results in the metabolic and stress assays. To determine interaction between diets and oxygen a 2-way ANOVA was performed. In lifespan and starvation assays, the significance was determined by comparing the survival curves with a log-rank (Mantel-Cox) test.

## Results

### Phenotypic Profile of Flies on High-fat Diet

**Figure 1:**
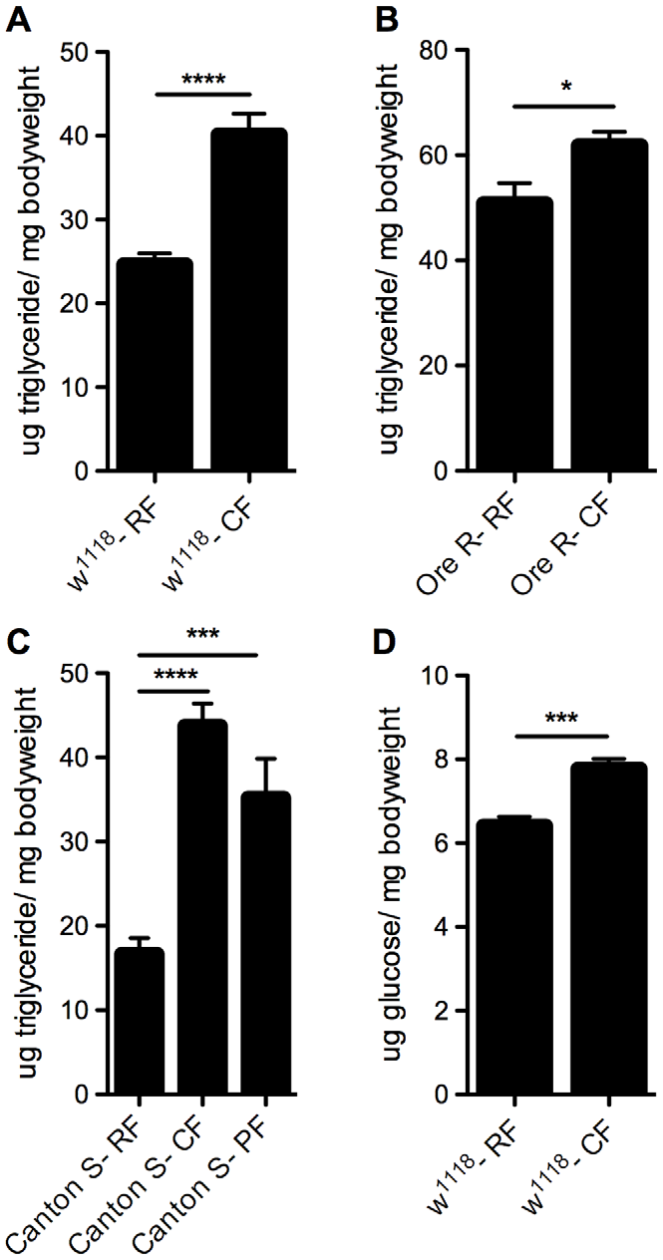
Changes in metabolic profile of flies on high-fat diet compared with regular diet. A–C) Triglyceride levels (per mg live weight) in whole-body homogenate of 10–12 day old adult female (A) *w^1118^*, (B) *Oregon-R* and (C) *Canton-S* flies after one week on diets in room air. Flies were on regular food (RF), high-fat coconut food (supplemented with 20% coconut oil, CF) or high-fat palm food (supplemented with 20% palm oil, PF). D) Glucose levels (per mg live weight) in whole-body homogenate of 10–12 day old adult female *w^1118^* flies, after one week on RF and CF diets in room air. Flies were homogenized in groups of five females: (A) n = 31 groups (155 flies), (B) n = 17 groups (85 flies), (C) RF n = 11 groups (55 flies), (D) n = 24 groups (120 flies). Error bars show SEM; * = p<0.05, *** = p<0.001, **** = p<0.0001.

To evaluate the effect of a diet high in fat on *Drosophila*, we placed female *w^1118^* flies on either a regular diet or food supplemented with coconut oil (rich in saturated fats) as 20% of the diet (see methods). After a week on these diets in normoxia, the flies on coconut oil supplemented food (CF) showed a large increase in whole-body triglyceride levels (p<0.0001) compared to those fed regular food (RF) (Figure 1A). To evaluate whether this increase seen with the CF diet was specific to the strain of fly or type of food, we also assayed the triglyceride levels of *Canton-S* and *Oregon-R* flies on RF and CF diets, as well as tested the effect of palm oil in the diet (PF) rather than coconut oil. Both fly strains responded in the same way as *w^1118^* flies, with increased triglyceride levels when on the CF diet, and a high-fat PF diet resulted in similar increases as CF (Figures 1B,C). Additionally we measured the whole-body free glucose levels in the *w1118* flies, as it is well known that obesity also affects glucose metabolism [15], [16]. Flies on the CF diet had significantly increased glucose levels (p<0.001) compared to those on the RF diet (Figure 1D).

**Figure 2:**
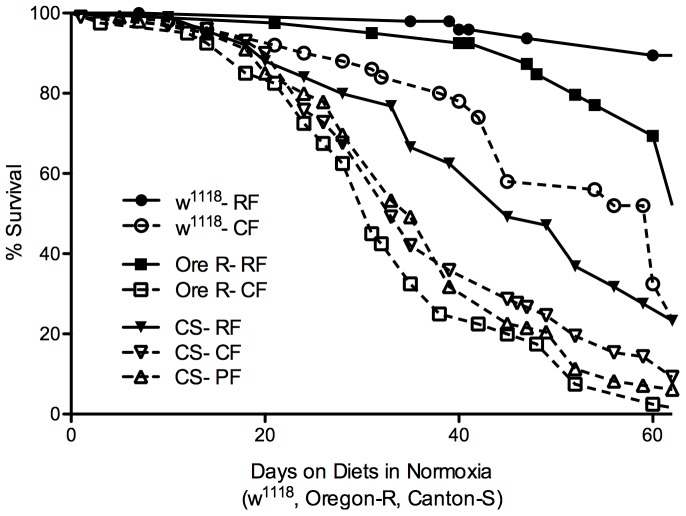
Altered lifespan due to high-fat diet. Adult female *w^1118^*, *Ore-R*, and *Canton-S* flies (d3-5, n = 50 per diet) were placed on regular (RF) and high-fat (CF) diets in room air. Additionally one group of *Canton-S* flies was placed on a high-fat diet using palm oil (PF). Flies were counted daily, with the number alive recorded. There was a significant difference between the regular and high-fat diet curves in each comparison; *w^1118^* RF vs. CF p<0.0001, *Ore-R* RF vs. CF p<0.0001, *Canton-S* RF vs. CF p<0.001, RF vs. PF p<0.0001 (Log-rank test).

The elevated triglyceride levels seen in obesity are often correlated with a decreased lifespan, both in humans [17] and *Drosophila* [18], [19]. We found that the high-fat diet had a detrimental effect on lifespan, as the lifespan of *w1118* flies on a CF diet was significantly shortened compared to RF flies (p<0.0001) when given full access to food (Figure 2). This significant decrease was also seen in the *Oregon-R* flies on CF diets (p<0.0001) and *Canton-S* flies on CF (p<0.001) or PF (p<0.0001) diets when compared to RF flies (Figure 2). Not surprisingly, under starvation conditions the *w1118* CF flies were more resistant to the stress of starvation than flies on RF diet and survived longer (p<0.05) (Figure S1A).

**Figure 3:**
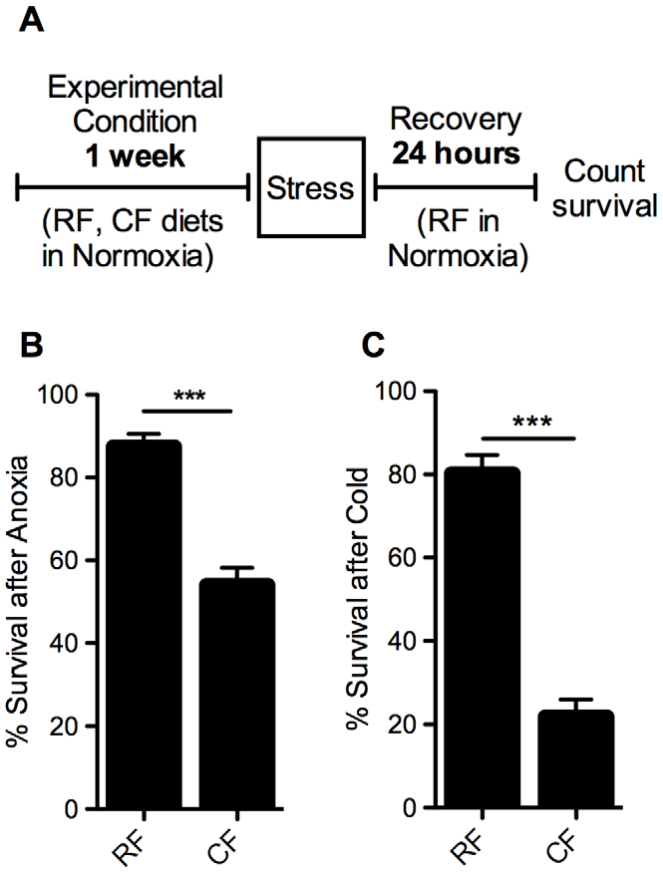
Decreased tolerance to acute stress in flies on high-fat diet. A) Diagram of experimental paradigm for stress assays. Adult female *w^1118^* flies were placed on regular (RF) or high-fat (CF) diets in room air for one week, after which they were stressed for 2 hours in B) anoxia (0% oxygen) or C) extreme cold (−5°C water bath). After recovering for 24 hours on regular food in room air, survival was counted as number of flies alive. Flies were tested in groups of 15 females per vial: (B) n = 18 groups (270 flies), (C) n = 16 groups (240 flies). Error bars show SEM; *** = p<0.001.

Since lifespan can be an indication of the ability of an organism to tolerate stress [20], we also tested stress resistance in these flies to cold and to anoxia (Figure 3A). The flies on the CF diet showed a marked decrease in tolerance to these two acute stresses. After 2 hours in anoxia (0% oxygen), nearly 90% of flies on the RF diet were able to recover during the subsequent 24 hours, compared with less than 60% of flies on the CF diet (Figure 3B). After exposure to 2 hours in −5°C, flies on a CF diet survived significantly less than their RF counterparts, with just a quarter of the RF survival rate (Figure 3C).

### Effects of Intermittent or Constant Hypoxia on Flies on a High-fat Diet

Similar to the evaluation of flies on the RF and CF diets in normoxia, flies were placed on the diets and immediately put in a hypoxic chamber in either intermittent (IH) or constant hypoxia (CH) for 7 days. IH and CH have been shown to have differential effects [21]–[23] and alter expression of different genes [12], so it was important to separately evaluate the response of the flies on the diets in both hypoxia conditions.

**Figure 4:**
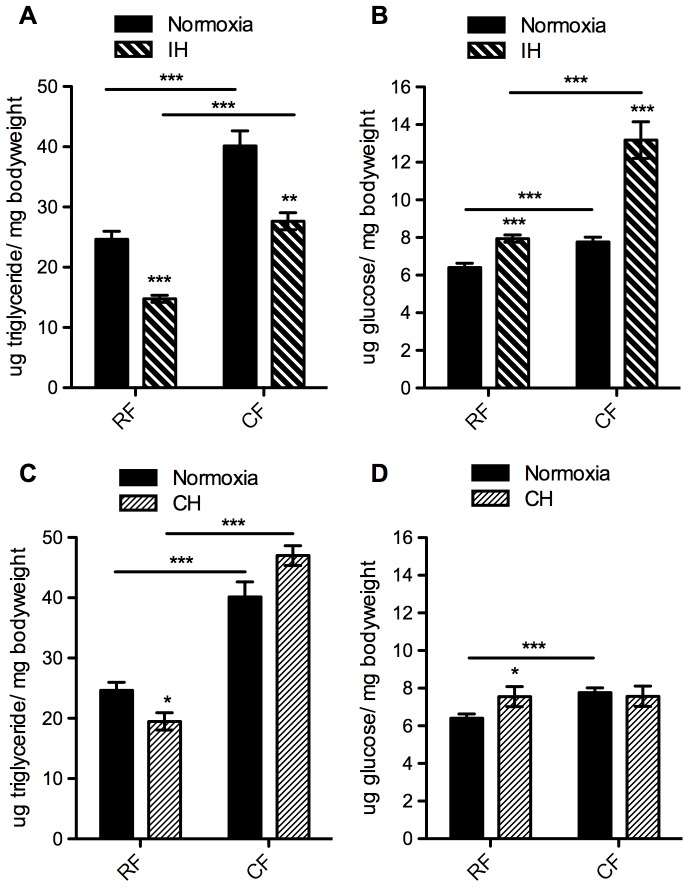
Changes in metabolic profile due to high-fat diet and hypoxia. A, C) Triglyceride levels (per mg live weight) and B, D) glucose levels (per mg live weight) were assayed in whole-body homogenate of 10–12 day old adult female *w^1118^* flies. Measurements were taken after one week on diets in normoxia (solid bars, A–D), intermittent hypoxia (IH, pattern bars, A and B) or constant hypoxia (CH, pattern bars, C and D). During that week, flies were on diets of either regular (RF) or high-fat diet (CF). Flies were homogenized in groups of five females: normoxia triglyceride (TG) n = 31 (155 flies), glucose n = 24 groups (120 flies); IH TG n = 17 (85 flies), glucose n = 18 groups (90 flies); CH TG n = 20 (100 flies), glucose n = 12 groups (60 flies). Error bars show SEM; * = p<0.05, ** = p<0.01, *** = p<0.001.

The alterations in the metabolic profile seen in normoxia were also present in both IH and CH, with increased TG levels in flies on the coconut-supplemented food (p<0.001) (Figure 4A, C). Although the difference in TG levels due to diet was persistent, there was also a difference in TG levels between flies on the same diet, depending on the oxygen condition. When triglyceride levels were compared between hypoxic and normoxic conditions in flies on the same diet, IH was associated with lower TG levels in the flies on both the RF and the CF diet (Figure 4A). There was no significant interaction between diet and oxygen level, so IH was not causing the flies to respond differently to the diet in terms of TG, but rather just causing an overall decrease regardless of diet. On the other hand, CH provided a very different response. While the TG levels in flies on the RF diet significantly decreased in CH compared to normoxia, the TG levels in flies on the CF diet increased (Figure 4C). The interaction between oxygen and diet was significant (p<0.01), indicating that being in constant low oxygen (i.e. in CH) affected flies on the CF diet differently than the RF diet.

**Figure 5:**
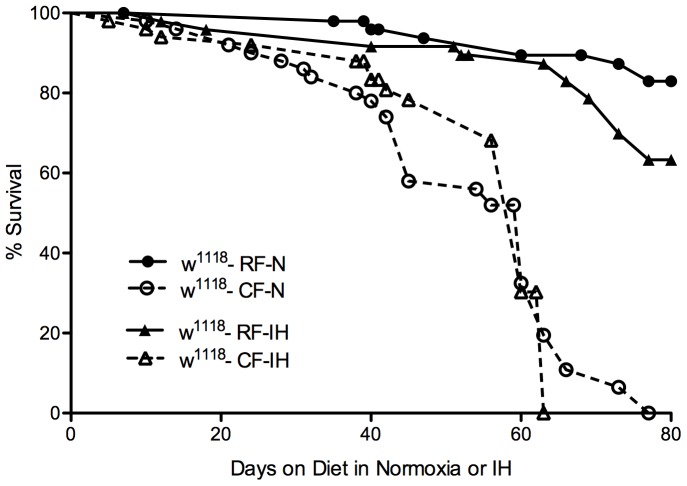
Altered lifespan due to high-fat diet and hypoxia. Adult female *w^1118^* flies (d3-5) were placed on regular (RF) and high-fat (CF) diets in normoxia (also shown in Figure 2) or intermittent hypoxia (IH) (n = 50 flies per diet). Flies were counted daily, with the number alive recorded. There was a significant difference between the regular and high-fat diet curves in each comparison, as well as between normoxia and IH when flies were on a regular diet; *w^1118^* RF-Normoxia vs. CF-Normoxia p<0.0001, *w^1118^* RF-IH vs. CF-IH p<0.0001, *w^1118^* RF-N vs. RF-IH p<0.05 (Log-rank test).

As in normoxia, glucose levels were significantly increased in flies on the CF diet compared to the RF diet when exposed to IH (Figure 4B). However, the flies in IH showed a much larger increase than those in normoxia, indicating that there was a significant interaction between diet and IH when the regulation of glucose levels was considered. Flies exposed to CH showed no significant difference in glucose levels between diets, although there was a slight increase in glucose levels between normoxia and CH in flies on the RF diet (Figure 4D). Similar to results in normoxia, flies on the CF diet in IH were more resistant to starvation than those on RF (p<0.0001) (Figure S1B) and had a significantly shortened lifespan (p<0.0001) (Figure 5). A comparable lifespan difference was seen in flies in constant hypoxia.

**Figure 6:**
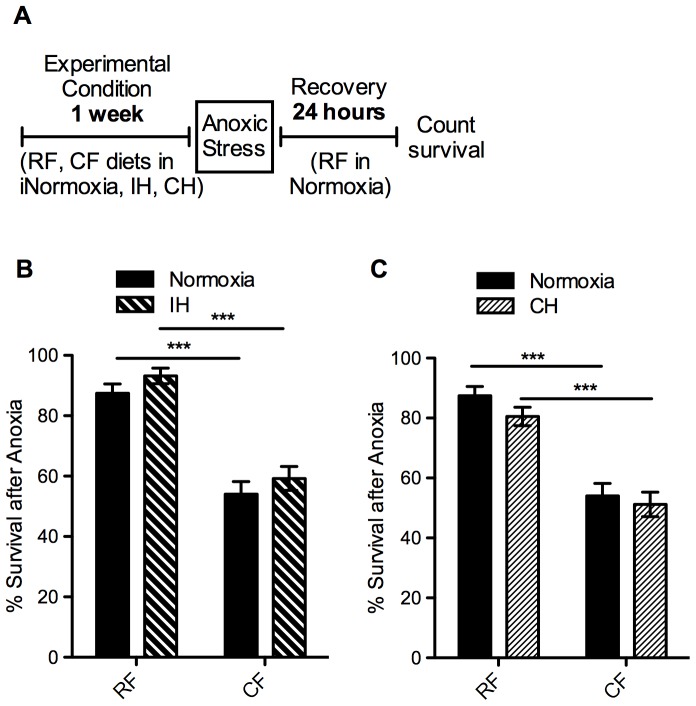
Anoxia tolerance unaltered by hypoxia exposure. Effect of prior exposure to intermittent hypoxia (IH) or constant hypoxia (CH) on survival of flies after 2 hours in anoxia (0% oxygen) was measured. A) Diagram of experimental paradigm for anoxia assay following hypoxia exposure. Adult female *w^1118^* flies (10–12 days old) were placed on regular (RF) or high-fat (CF) diets in normoxia (B, C solid bars), IH (B, pattern bars), or CH (C, pattern bars) for one week prior to anoxia assay. Flies were removed from hypoxia and immediately assayed for tolerance to 2 hours of acute anoxia. After recovering for 24 hours on RF in room air, survival was measured as number of flies alive. Flies recovered in groups of 15 flies per vial: normoxia n = 18 groups (270 flies); IH n = 13 groups (195 flies); CH n = 17 groups (255 flies). Error bars show SEM; *** = p<0.001.

**Figure 7:**
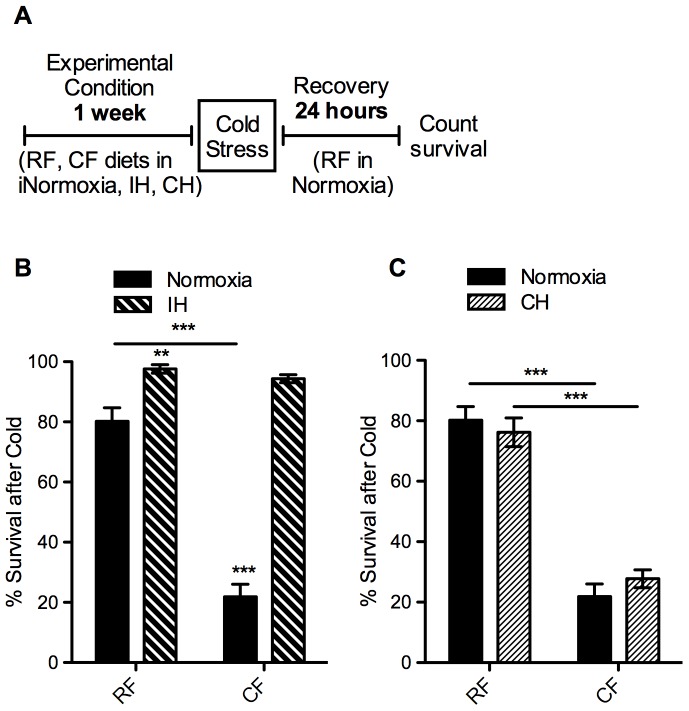
Cold survival altered by intermittent hypoxia, but not constant hypoxia. Effect of prior exposure to intermittent hypoxia (IH) or constant hypoxia (CH) on survival of flies after 2 hours in extreme cold (−5°C water bath) was measured. A) Diagram of experimental paradigm for cold stress assay following hypoxia exposure. Adult female *w^1118^* flies (10–12 days old) were placed on regular (RF) or high-fat (CF) diets in normoxia (B, C solid bars), IH (B, pattern bars), or CH (C, pattern bars) for one week prior to cold stress assay. Flies were removed from hypoxia and immediately assayed for tolerance to acute cold stress. After recovering for 24 hours on RF in room air, survival was measured as number of flies alive. Flies were tested in groups of 15 flies per vial: normoxia n = 17 groups (255 flies); IH n = 14 groups (210 flies); CH n = 22 groups (330 flies). Error bars show SEM; ** = p<0.01, *** = p<0.001.

The response to stress also varied with the two hypoxic treatments. Flies on a CF diet in IH and CH had decreased survival after anoxic stress when compared to RF, similar to the response seen in normoxia (Figure 6). In contrast, survival after cold stress was very much affected by the hypoxia paradigm. There was a marked increase in survival after cold stress even in flies on the RF diet in IH compared to normoxia. Flies on the CF diet in IH saw a remarkable change in response, with nearly full survival in IH compared to just over 20% survival in normoxia (Figure 7A, B). There was no difference in survival between diets in the IH flies, but there was a highly significant interaction (p<0.0001) in how the oxygen condition affected the flies on the two diets. This difference is not seen in CH, as the flies responded similarly as in normoxia, with a decreased survival in the CF flies after cold stress (Figure 7C).

**Figure 8:**
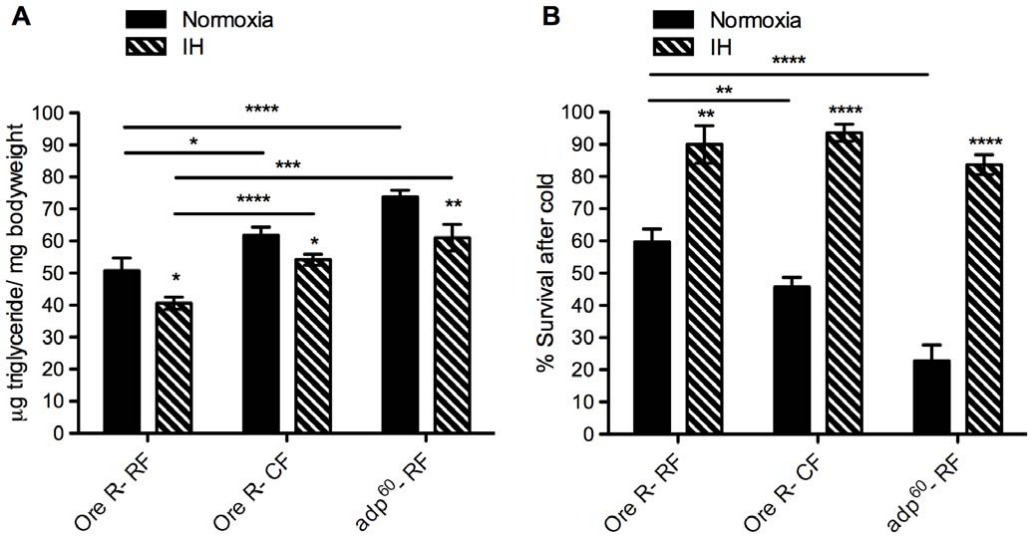
Changes due to intermittent hypoxia also seen in genetic mutant model of obesity. A genetic mutant model of obesity, *adipose^60^ (adp^60^)*, was assayed in normoxia and intermittent hypoxia (IH) with the *Oregon-R (Ore-R)* strain as control. Adult female *adp^60^* flies (10–12 days old) were placed on the regular (RF) diet and adult *Ore-R* females were placed on RF and high-fat (CF) diets. The flies were kept in normoxia (solid bars) or IH (pattern bars) for one week. Following that week, the flies were assayed for A) triglyceride levels (per mg live weight) in whole-body homogenate and B) survival of flies after 2 hours in extreme cold (−5°C water bath), measured 24 hours after return to room air. For triglyceride determination, flies were homogenized in groups of five females, normoxia: *Ore-R* n = 17 groups (85 flies), *adp^60^* n = 20 groups (100 flies); IH: *Ore-R* n = 13 groups (65 flies), *adp^60^* n = 15 groups (75 flies). In the cold assay, flies were tested in groups of 15 flies per vial: normoxia *Ore-R* n = 13 groups (65 flies), *adp^60^* n = 12 groups (60 flies); IH *Ore-R* n = 8 groups (40 flies), *adp^60^* n = 11 groups (55 flies). Error bars show SEM; * = p<0.05, ** = p<0.01, *** = p<0.001, **** = p<0.0001.

To further describe the effect of IH on cold stress recovery, it was important to test an additional paradigm causing increased fat storage. In order to do this, we utilized the well-characterized genetic mutant *adipose^60^* (*adp^60^*). On a regular diet, these mutants have significantly higher triglyceride levels than wildtype flies, and even surpass the triglyceride level of wildtype flies on a high-fat diet. These triglyceride levels drop slightly after IH, as they do in the wildtype flies on both diets (Figure 8A). Importantly, IH almost fully restores cold stress survival in the *adp^60^* mutant flies, as was seen with wildtype flies on both diets (Figure 8B).

**Figure 9:**
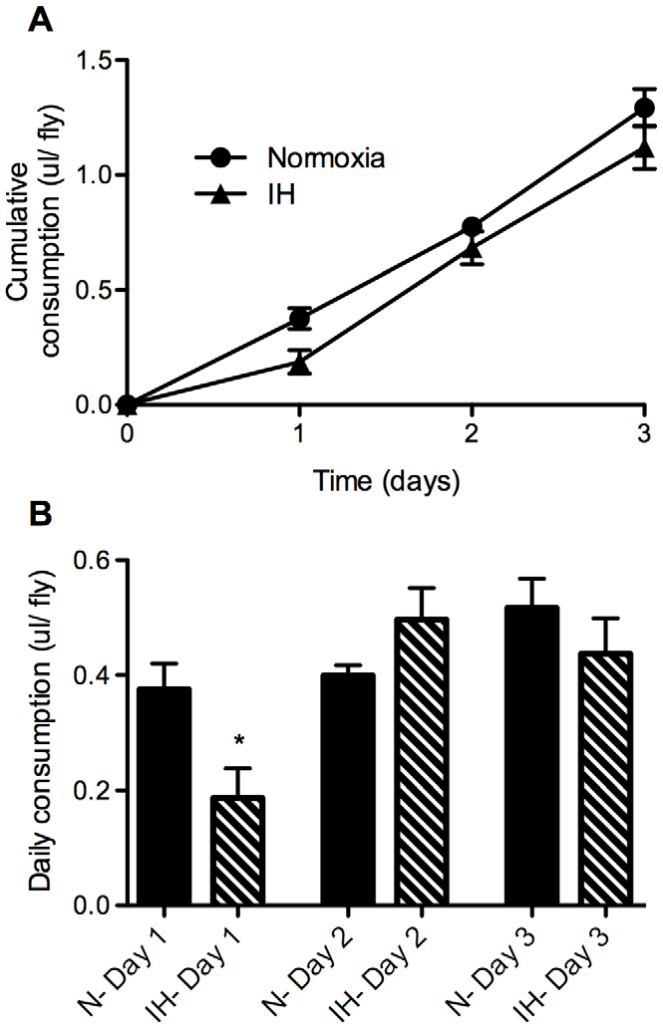
Similar food consumption observed in normoxia and intermittent hypoxia. Adult female *w^1118^* flies (3–5 days old) were placed on regular (RF) for 5 days in normoxia or intermittent hypoxia (IH). Following that time, flies were transferred to the CAFE vials and returned to normoxia or IH. In the CAFE setup, flies had access to water but had to obtain their food out of a capillary tube through the top of the vial. Measurements of the change in liquid food level in the capillary tube allowed for determination of the total food consumed A) cumulatively over 3 days and B) on a daily basis. Flies were tested in groups of 5, n = 14 groups (70 flies). Error bars show SEM; * = p<0.05.

Given the consistent decrease in triglyceride levels in flies on both diets in IH, we felt it was important to determine whether this change was due to an altered level of food consumption. We adapted the CAFE set-up described in Ja et al [14] for normoxic and IH conditions, using flies previously on RF in normoxia or IH. After an initial decrease in food consumption in IH flies on the first day of CAFE measurement, there appears to be no significant difference between the slopes of the cumulative consumption, as determined by a Deming (Model II) Linear Regression (Figure 9A) and no significant differences on the 2^nd^ and 3^rd^ day in the daily food consumption between flies in normoxia as compared to IH (Figure 9B).

## Discussion

We have generated a model showing the detrimental effects of a high-fat diet on lifespan and stress tolerance in *Drosophila*. Flies on a diet supplemented with coconut oil (or palm oil) significantly increased their triglyceride and glucose levels and shortened their lifespan. This diet, high in saturated fat, not only hindered flies from living as long as others on the regular diet, but it also greatly diminished their tolerance to acute stresses. When challenged with either anoxia or severe cold, few flies on the high-fat diet survived, while most of those on the regular diet did. Conversely, under starvation conditions the flies on the high-fat diet showed increased resistance, due to their increased triglyceride and energy stores.

The link between hypoxia and metabolism has been demonstrated in models from flies and mice to humans [24]–[28]. Intermittent hypoxia in particular has been strongly implicated as having a role in altering glucose and lipid metabolism. In rats, IH has been shown to lead to oxidative stress, lipid peroxidation, neuronal apoptosis and up-regulation of stress responsive proteins [29], [30]. Chronic IH has been shown to cause insulin resistance and glucose intolerance in obese leptin-deficient mice [25], [31] as well as inducing hyperlipidemia in lean mice [25]. Although studies in humans are more limited, experimental evidence supports a detrimental effect of hypoxia on metabolism, with diminished insulin sensitivity in subjects after just 30 minutes of hypoxia exposure [24]. Our model presents an essential first step in understanding the *Drosophila* response to a high-fat diet and modulation of this response by IH.

When investigating the phenotype of flies on a high-fat diet, both independently and in conjunction with hypoxia, we made two important observations and pose here two interesting questions that arise from these results. First, since the coconut oil diet led to a decreased survival following cold stress, our question is: how does this high-fat diet cause decreased survival in this stressful condition? Second, and of interest, IH led to rescuing flies from decreased survival after a severe cold stress; how does IH allow flies to fully recover from this stress?

Normally, cells convert excess non-esterified free fatty acids into triglycerides, which is why the coconut oil diet led to increased levels of TG in our model. With this excess storage of triglycerides, we showed that flies had decreased tolerance to stresses such as anoxia and cold. Keeping in mind the diminished recovery from cold stress in flies fed coconut oil, it is likely that the high-fat diet alters energy and metabolic pathways in such a way that flies are unable to activate some of the key mechanisms needed to recover from the cold stress. This assumes that genes are activated not only during the cold exposure itself but also during recovery from cold. Indeed, Clark and Worland have demonstrated just that [32]. For example, it is known that heat shock proteins, which are well characterized in their response to stress, are important in recovery. Newly discovered genes in this area, such as *starvin* and *frost* [33]–[36], have also been implicated. While its exact function in recovery from cold stress is not known, starvin is up-regulated in the recovery phase following cold stress and is believed to be a co-chaperone regulating the Hsp70 complex during recovery from cold. A mucin-like protein, frost is thought to play a role in protecting against oxidative stress and maintaining membrane integrity, thus aiding in the ability to recover from cold stress. We therefore hypothesize that flies on the CF diet were unable to activate mechanisms such as these, jeopardizing their recovery and survival from cold.

The inability of flies on the high-fat diet to recover from the stress of cold was completely rescued when these flies had been in intermittent hypoxia prior to the cold. Since this rescue of survival did not occur in flies in CH, these data would indicate that IH specifically, and not just hypoxia in general, is necessary for this rescue phenotype. It appears that intermittent hypoxia alters processes in such a way to override the negative effect of increased triglycerides, whether from a high-fat diet or as a result of a genetic mutation (*adp^60^*). One possible explanation could involve the increase in glucose levels seen in flies on both RF and CF diets following IH. Sugars such as glucose and trehalose are considered cryoprotective molecules and have been implicated in maintenance of cell function at low temperatures [37], [38]. With this accumulation of free glucose during IH, the fly may be better prepared to survive the cold stress. Another potential mechanism may be related to other alterations in gene expression. For example, we have previously shown that IH induces expression of genes important in transport and defense, including the high affinity inorganic phosphate: sodium symporter, l(2)08717 [12]. Ion transport appears to play an important role in survival to extreme cold, as lower temperatures can lead to decreased ion pump activity, decreased membrane fluidity and inhibited ion channel gating [32], [39]. If exposure to IH up-regulated expression of genes is important in ion transport, we speculate here that this IH is tantamount to a pre-conditioning of the flies to stress, allowing them to resist the effects of the high-fat diet in the survival from cold stress.

In summary, we found that flies on a high-fat diet have a drastically worsened phenotype, with decreased resilience to stress and decreased survival compared to those on a regular diet, and this phenotype is altered by exposure to hypoxia. With significantly increased triglyceride and glucose levels in normoxic conditions, flies on a high-fat diet have a shortened lifespan and decreased tolerance to stress. While the triglyceride and glucose levels are altered with hypoxia, the most interesting change was seen when exposure to IH appeared to rescue survival after cold stress. The detrimental effects of a high-fat diet are clear in this *Drosophila* model, and there appears to be both deleterious and compensatory cross talk occurring between hypoxia and the high-fat diet. With the climbing global obesity levels and lack of understanding of the interaction between obesity and hypoxia, developing an animal model that lends itself to an investigation of this interaction becomes vital.

## Supporting Information

Figure S1 **Altered starvation resistance due to high-fat diet.** Adult female *w^1118^* flies (3–5 days old) were placed on regular (RF) or high-fat (CF) diets in A) normoxia (N) or B) intermittent hypoxia (IH) for one week (n = 110 flies per group). Following that week, flies were transferred to plastic vials without food, but with access to water. Flies were kept in room air and counted every 4–6 hours, with the number alive recorded. There was a significant difference between the survival curves; A) p = 0.02, B) p<0.0001 (Log-rank test).
(TIFF)
