# Supplementary figures and images for: Role of High-Fat Diet in Stress Response of Drosophila
Source: PLoS One. 2012 Aug 1;7(8):e42587. doi: 10.1371/journal.pone.0042587 (PMC3411628; doi:10.1371/journal.pone.0042587)

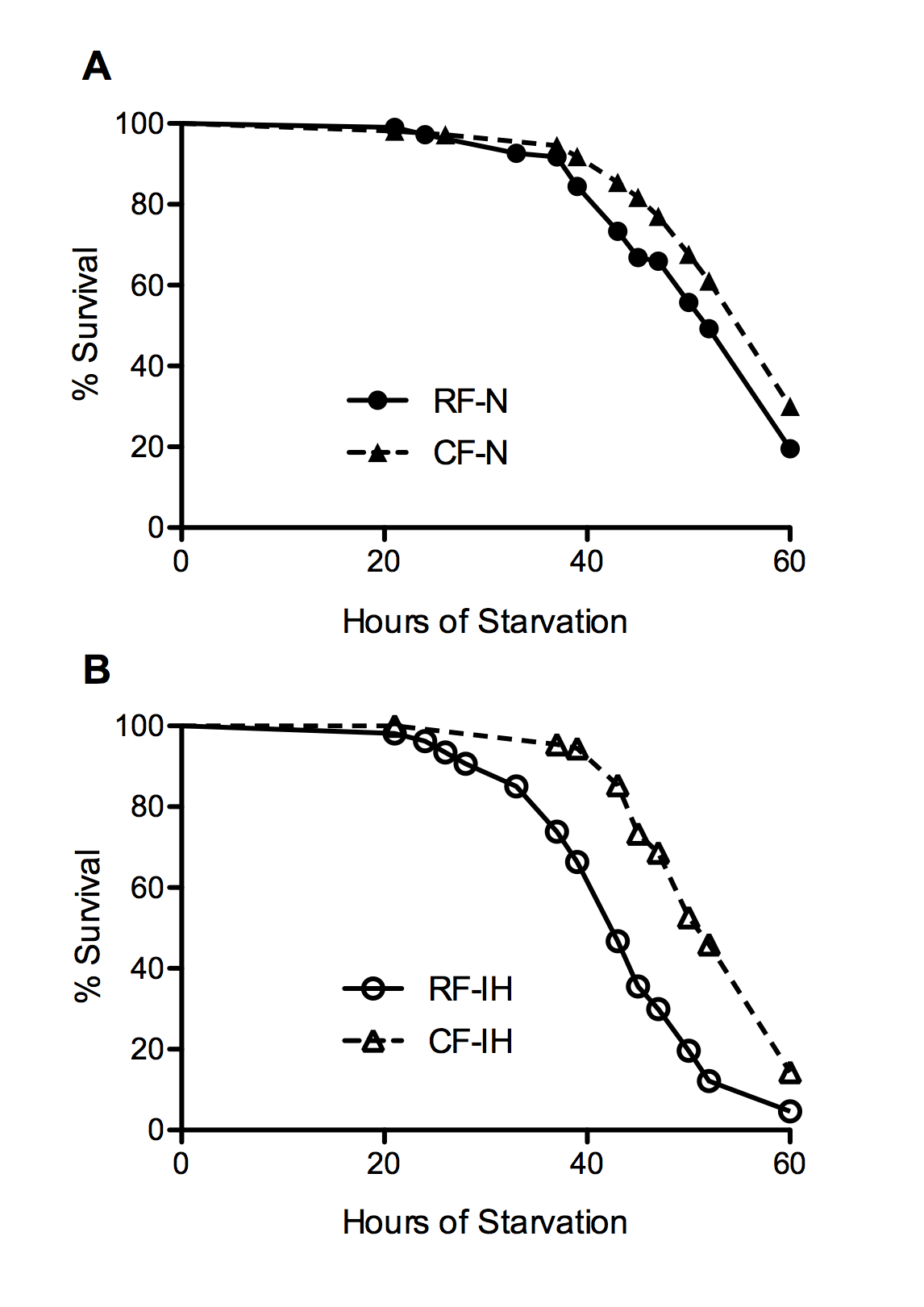

Supplement: Figure S1 — Altered starvation resistance due to high-fat diet. Adult female w1118 flies (3–5 days old) were placed on regular (RF) or high-fat (CF) diets in A) normoxia (N) or B) intermittent hypoxia (IH) for one week (n = 110 flies per group). Following that week, flies were transferred to plastic vials without food, but with access to water. Flies were kept in room air and counted every 4–6 hours, with the number alive recorded. There was a significant difference between the survival curves; A) p = 0.02, B) p<0.0001 (Log-rank test). (TIFF) [file pone.0042587.s001.tiff]
